# In vitro cellular testing of strontium/calcium substituted phosphate glass discs and microspheres shows potential for bone regeneration

**DOI:** 10.1002/term.2796

**Published:** 2019-02-17

**Authors:** Uresha Patel, Laura Macri‐Pellizzeri, Kazi M. Zakir Hossain, Brigitte E. Scammell, David M. Grant, Colin A. Scotchford, Alex C. Hannon, Andrew R. Kennedy, Emma R. Barney, Ifty Ahmed, Virginie Sottile

**Affiliations:** ^1^ Faculty of Engineering University of Nottingham Nottingham UK; ^2^ Wolfson STEM Centre, School of Medicine University of Nottingham Nottingham UK; ^3^ Orthopaedics and Trauma Group, Division of Rheumatology, Orthopaedics, and Dermatology, School of Medicine University of Nottingham Nottingham UK; ^4^ ISIS Facility Rutherford Appleton Laboratory, Chilton Didcot UK; ^5^ Faculty of Science and Technology Lancaster University Lancaster UK

**Keywords:** bone regeneration, calcium phosphate glass, stem cells, strontium

## Abstract

Phosphate‐based glasses (PBGs) are ideal materials for regenerative medicine strategies because their composition, degradation rates, and ion release profiles can easily be controlled. Strontium has previously been found to simultaneously affect bone resorption and deposition. Therefore, by combining the inherent properties of resorbable PBG and therapeutic activity of strontium, these glasses could be used as a delivery device of therapeutic factors for the treatment of orthopaedic diseases such as osteoporosis. This study shows the cytocompatibility and osteogenic potential of PBGs where CaO is gradually replaced by SrO in the near invert glass system 40P_2_O_5_·(16‐x)CaO·20Na_2_O·24MgO·xSrO (x = 0, 4, 8, 12, and 16 mol%). Direct seeding of MG63 cells onto glass discs showed no significant difference in cell metabolic activity and DNA amount measurement across the different formulations studied. Cell attachment and spreading was confirmed via scanning electron microscopy (SEM) imaging at Days 3 and 14. Alkaline phosphatase (ALP) activity was similarly maintained across the glass compositions. Follow‐on studies explored the effect of each glass composition in microsphere conformation (size: 63‐125 μm) on human mesenchymal stem cells (hMSCs) in 3D cultures, and analysis of cell metabolic activity and ALP activity showed no significant differences at Day 14 over the compositional range investigated, in line with the observations from MG63 cell culture studies. Environmental SEM and live cell imaging at Day 14 of hMSCs seeded on the microspheres showed cell attachment and colonisation of the microsphere surfaces, confirming these formulations as promising candidates for regenerative medicine strategies addressing compromised musculoskeletal/orthopaedic diseases.

## 1 INTRODUCTION

Phosphate‐based glasses (PBGs) are highly suited biomaterials to deliver therapeutic ions, particularly for bone regeneration, because their main constituents (i.e., calcium, phosphate, sodium, and magnesium) are found to occur in the inorganic phase of natural bone (Jones & Clare, 2012). The ability to control their dissolution rates, and thus the release of ions, serves as a highly attractive route to direct the biological response for the repair and regeneration of both hard and soft tissues (Hoppe, Guldal, & Boccaccini, 2011). Furthermore, the geometry of PBG can be varied by means of fibre drawing (Ahmed, Collins, Lewis, Olsen, & Knowles, 2004; Sharmin, Rudd, Parsons, & Ahmed, 2016), scaffold production (Jones et al., 2010), and microsphere manufacture (Jones et al., 2010; Lakhkar et al., 2012), making them an extremely versatile and tuneable material.

A particular ion of interest is strontium (Sr), especially for use in hard‐tissue repair applications; it has been investigated for incorporation and/or substitution into a vast array of materials such as organic polymers, ceramics, glasses, composites, and alloys (Han et al., 2016; Liu, Li, Ji, & Jia, 2013; Mushahary et al., 2016; Park, Kang, & Hanawa, 2016; Ray et al., 2016; Schumacher & Gelinsky, 2015; Wu, Lin, Yang, & Lee, 2015). Strontium is also of clinical importance because it has been shown to promote bone formation via stimulation of pre‐osteoblastic cells replication and to reduce bone resorption via inhibition of osteoclast formation and activity (Canalis, Hott, Deloffre, Tsouderos, & Marie, 1996). In the past, strontium ranelate was administrated to osteoporotic patients, due to its anabolic activity and antiresorptive effect on bone tissue (Ammann, 2005), as the imbalance of bone deposition and resorption characterises osteoporosis (Blake & Fogelman, 2006). However, since 2013, the European Medicine Agency has restricted the use of strontium ranelate for the treatment of severe osteoporosis in postmenopausal women and adult men at high risk of fracture due to increased cardiovascular risks (National Institute for Health and Care Excellence (NICE), 2011). Therefore, investigation into alternative administration routes such as controlled and localised delivery of the ion, as opposed to systemic delivery, could be very beneficial.

Substitution of calcium for strontium in biomaterials (e.g., orthopaedic cements and bioactive glasses) has been of interest as both play a similar chemical role because they are divalent cations with a comparable ionic size (Ca^2+^ 1.00 Å and Sr^2+^ 1.18 Å) and are accumulated in bone. Due to the ease of substitution of these two cations within a glass system, as well as the positive influence strontium can have on bone metabolism, the use of this ion for bone tissue engineering and regenerative medicine strategies constitutes an area of growing interest (Abou Neel et al., 2009; Al Qaysi et al., 2015; Lee, Obata, Brauer, & Kasuga, 2015). Several studies have investigated the behaviour of cells such as osteoblast‐like cells and mesenchymal stem cells towards strontium‐containing biomaterials (Aina et al., 2013; Lin et al., 2013; Ni, Shu, Huang, Lu, & Pan, 2012; Santocildes‐Romero et al., 2015). For instance, Sr‐substituted hydroxyapatite nanocrystals were shown to dose dependently promote higher levels of alkaline phosphatase (ALP) activity, collagen type‐I, and osteocalcin production in comparison with pure hydroxyapatite in the osteoblast‐like MG63 cell line (Capuccini et al., 2009). Investigation into the effects of Sr‐containing PBG has also been reported; for example, human osteosarcoma cells showed higher degree of adhesion to PBG with 1 and 3 mol% SrO substitution in comparison with the 5% and SrO‐free control (Abou Neel et al., 2009).

The aim of this study was to investigate the cytocompatibility of near invert phosphate glasses where calcium was eventually fully substituted for strontium on a gradual basis in a previously reported glass system 40P_2_O_5_·(16‐x)CaO·20Na_2_O·24MgO·xSrO (x = 0, 4, 8, 12, and 16 mol%; Patel et al., 2017). All formulations were manufactured as discs and microspheres and tested for their ability to support adhesion, proliferation, and osteogenic differentiation using human osteoblast‐like MG63 cells and human mesenchymal stem cells (hMSCs), respectively, in order to investigate their potential use for bone repair applications via two clinically relevant cell types.

## 2 MATERIALS AND METHODS

### 2.1 PBG sample production

The following precursors were used to fabricate glass rods of the compositions shown in Table 1: P_2_O_5_, NaH_2_PO_4_, CaHPO_4_, MgHPO_4_·3H_2_O, and SrCO_3_ (Sigma Aldrich, UK). The weighed precursors were thoroughly mixed and then heated in a 5% Au/95% Pt crucible at 350°C for 30 min to dehydrate and remove CO_2_ before placing the crucible into a furnace heated to 1150°C for 90 min. The melts were poured into preheated graphite moulds (10°C) above T*g* of each sample and annealed for 1 hr, followed by slow cooling to room temperature overnight. Each rod was then cut into discs of 9 mm × 2 mm using a diamond saw using methanol as a lubricant agent. Sterilisation of the discs was carried out via UV light exposure for 1 hr on either side.

**Table 1:** Compositional information, glass transition temperature, and glass code of each formulation

Glass code	P_2_O_5_ (mol%)	CaO (mol%)	Na_2_O (mol%)	MgO (mol%)	SrO (mol%)	*T*_g_ (±1°C)
P40	40	16	20	24	—	450
Sr4	40	12	20	24	4	447
Sr8	40	8	20	24	8	447
Sr12	40	4	20	24	12	447
Sr16	40	—	20	24	16	443

### 2.2 Glass microsphere production

The quenched glass samples were ground using a planetary zirconia ball mill (Retsch Planetary Mill PM100), operated for 2 min at 500 rpm. Glass powder was separated into particle size in the range 63–125 μm using stainless steel sieves (VWR International, UK). The ground particles were fed through a flame spheroidisation apparatus consisting of (a) a powder feeder, (b) an oxy/acetylene thermal spray gun (MK74 Thermal Spray gun Metallisation Ltd., UK), and (c) a series of collection vessels. Microspheres were collected from the collection vessel furthest away from the spray gun. Microspheres of all compositions were sterilised by washing twice in 70% EtOH for 15 min followed by complete evaporation at room temperature in a sterile environment.

### 2.3 Cell culture

For cell experiments, materials were purchased from ThermoFisher Scientific (UK) unless otherwise stated.

The osteoblast‐like cell line MG63 (obtained from European collection of cell cultures ‐ ECACC) was seeded on each disc at a density of 4 × 10^4^ cells/cm^2^ and cultured using Dulbecco's Modified Eagle Media supplemented with 10% fetal calf serum, 2% HEPES Buffer, 2% antibiotics–antimycotic agents, 1% l‐glutamine, 1% non‐essential amino acids and 0.85 mM of ascorbic acid (Sigma Aldrich, UK) at 37°C and 5% CO_2_ in a 48‐well plate. Tissue culture plastic (TCP) was employed as control of conventional culture method.

For the experiments with the PBG microspheres, 2 × 10^4^ GFP‐labelled hMSCs (Harrison et al., 2017) were seeded onto 10‐mg sterile microspheres in low‐adherent 48‐well plate previously coated with 1% *w*/*v* solution of poly(2‐hydroxyethyl methacrylate) (poly‐HEMA, Sigma Aldrich) and ethanol 95%. Cells were cultured during 14 days at 37°C and 5% CO_2_ in standard cell (SC) culture medium (low glucose Dulbecco's Modified Eagle Media supplemented with 10% fetal calf serum, 1% penicillin and streptomycin, 1% l‐glutamine, and 1% of non‐essential amino acids). For both cell types, medium was refreshed every 48 hr.

### 2.4 Cell metabolic activity

Cell metabolic activity of MG63 cells cultured on PBG discs and TCP was evaluated at Days 1, 3, 7, and 14 using Alamar Blue assay. Briefly, 1 ml of Alamar Blue solution (1:9 Alamar blue:Hanks Balanced Salt Solution) was added to each well and incubated for 90 min at 37°C and 5% CO_2_ followed by further 10 min on a shaker at 150 rpm. For each disc, three aliquots of 100 μl were transferred to a 96‐well plate. FLx800 fluorescence microplate reader (BioTek Instruments Inc.) was used to measure fluorescence at 530‐nm excitation and 590‐nm emission wavelengths.

Cell metabolic activity of hMSCs was assayed using Presto Blue reagent at Days 2, 7, and 14 after seeding onto the microspheres according to the manufacturer's instructions. Briefly, a solution of SC medium supplemented with 10% of Presto Blue reagent was prepared, and 300 μl was added to the cells for 40 min at 37°C and 5% CO_2_. After the incubation, 250 μl of the solution was transferred to a clear bottom 96‐well plate; fluorescence measurement was performed in the microplate reader Infinite 200 (Tecan, CH) setting 560 nm and 590 nm as excitation and emission wavelengths, respectively.

### 2.5 Evaluation of DNA content

DNA content was evaluated in MG63 cells at Days 1, 3, 7, and 14 of culture on PBG discs and TCP control. Briefly, samples were washed three times with warm (37°C) PBS and immersed in 1 ml of deionised water. Samples were frozen‐thawed three times to lyse the cells and release nuclear content. Lysed samples were then thoroughly mixed using a vortex for 30–60 s, and 100 μl of each sample was aliquoted into a 96‐well plate. Hoechst 33258 stain solution was prepared by dissolving 1 mg of bisbenzimide stain in 1 ml of distilled water and diluted 1:50 in TNE buffer (10 mM Tris, 2 M NaCl, and 1 mM EDTA in deionised water, adjusted to pH 7.4); DNA standard curve was prepared using calf thymus DNA (Sigma, UK) diluted in TNE buffer. Each well was then topped with 100 μl of Hoechst 33258 stain and agitated using a plate shaker. Fluorescence was measured at 360 nm and 460 nm as excitation and emission wavelengths using FLx800 microplate fluorimeter (BioTek Instruments), respectively.

### 2.6 ALP activity

The Granutest 25 ALP assay (Randox, UK) was used to measure ALP activity in MG63 cells. Three aliquots of 50 μl of cell lysate (as prepared for DNA quantification assay in Section 3.4) were transferred to a 96‐well plate and topped with 50 μl of ALP substrate (*p*‐nitrophenyl phosphate 10 mM in diethanolamine buffer 1 mM at pH 9.8, with MgCl_2_ 0.5 mM). Plates were shaken gently for 5 min on a plate shaker, and absorbance was measured at wavelength of 405 nm using an FLx800 microplate colorimeter (BioTek Instruments).

ALP activity of hMSCs was assayed at Day 14 after seeding on the PBG microspheres. Briefly, live cells were washed twice with PBS and incubated with 300 μl of a solution of 1 mg/ml *p*‐nitrophenyl phosphate and 0.2 M Tris buffer (SIGMAFAST, Sigma‐Aldrich) prepared according to the manufacturer's instructions. ALP activity was monitored in the microplate reader (Tecan, CH) analysing the optical density at 405 nm performing 12 readings over 24 min. ALP solution was subsequently replaced with fresh SC medium, and cells returned to the incubator.

### 2.7 Cell imaging

At Days 3 and 14, MG63 cells seeded on PBG discs were washed three times with warm (37°C) PBS and fixed with 3% glutaraldehyde in 0.1 M sodium cacodlyated buffer for 30 min. Fixative was then replaced with 7% sucrose solution for 30 min. Specimens were again washed three times with 0.1 M cacodylate buffer for 5 min. Each sample was then coated with 1% osmium tetroxide in PBS for 45 min. This was followed by a dehydration process by incubating the samples in a graded ethanol series (20%, 30%, 40%, 50%, 60%, 70%, 80%, 90%, and 100% ethanol) for 5 min in each concentration and dehydrating the samples in 100% ethanol for a second time for 5 min. Samples were dried via hexamethyldisilazane, mounted onto a carbon scanning electron microscopy (SEM) stub and sputter‐coated with ~15 nm of platinum. Cells were visualised using a Philips XL30 scanning electron microscope operated at 10 kV.

Fluorescence live cell imaging of hMSCs seeded on the PBG microspheres was performed at Days 1 and 14 using a Juli FL Stage system and a Leica DM IRB microscope connected with a QICAM Fast 1394 camera, respectively. For environmental SEM (ESEM), hMSCs were fixed at Day 14 through a 10‐min incubation with 4% paraformaldehyde, washed twice with distilled water, and analysed using a FEI Quanta 650 ESEM microscope.

### 2.8 Statistical analysis

Three independent experiments were performed, and results are shown as mean ± standard error of mean. Statistical analysis was performed using Prism software package (version 7.01, GraphPad Software, San Diego, CA, www.graphpad.com). Two‐way analysis of variance was calculated followed by a Tukey's multiple comparison test. The mean difference was considered to be significant at 0.05 and 95% confidence interval.

## 3 RESULTS

### 3.1 Evaluation of MG63 cell growth on disc PBG formulations

**Figure 1:**
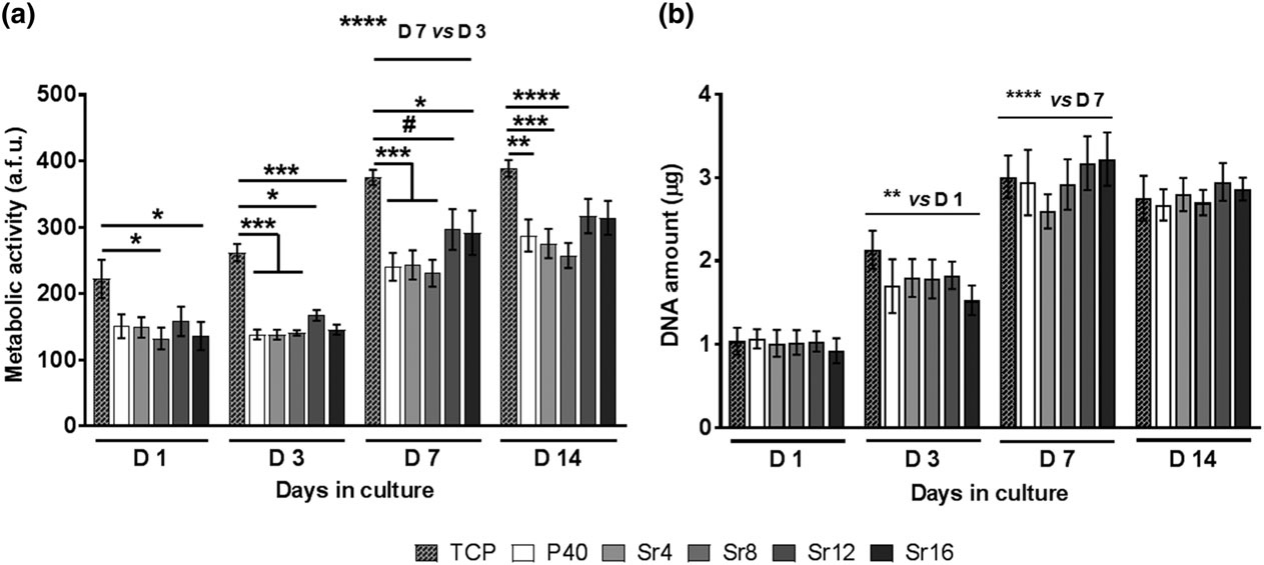
Quantification of metabolic activity (a) and DNA amount (b) in MG63 after 1, 3, 7, and 14 days of culture on phosphate‐based glass discs with varying Sr mol% and TCP. a.f.u.: arbitrary fluorescence units; TCP: tissue culture plate. Error bars represent standard error of mean; *n* = 15. ^*^ *p* < 0.05, ^**^ *p* < 0.01, ^***^ *p* < 0.001, ^****^ *p* < 0.0001, # *p* = 0.0594

In order to determine the cytocompatibility of glass samples with varying SrO content, the osteoblast‐like MG63 cell line was seeded onto the surface of glass discs, and the cell metabolic activity as well as DNA quantity was analysed at Days 1, 3, 7, and 14. Metabolic activity analysed using the Alamar Blue assay showed an increase in cell response for all compositions from Day 3, whereas no significant differences were detected between formulations at all the time points considered (*p* > 0.9). At Days 3 and 7, significantly greater values were observed for the TCP control in comparison with all glasses, whereas at Day 14, this difference was maintained only in comparison with P40, Sr4, and Sr8 (see Figure 1a).

Moreover, the quantification of total cell DNA amount showed a significant increase at each time point up to Day 7 (D1 vs. D3: *p* < 0.01; D7 vs. D3: *p* < 0.0001), followed by a plateau at Day 14. It is worth noting that no significant differences were observed between all formulations and the TCP control at each time point (*p* > 0.9; see Figure 1b).

### 3.2 Evaluation of hMSC cell growth on microsphere PBG formulations

**Figure 2:**
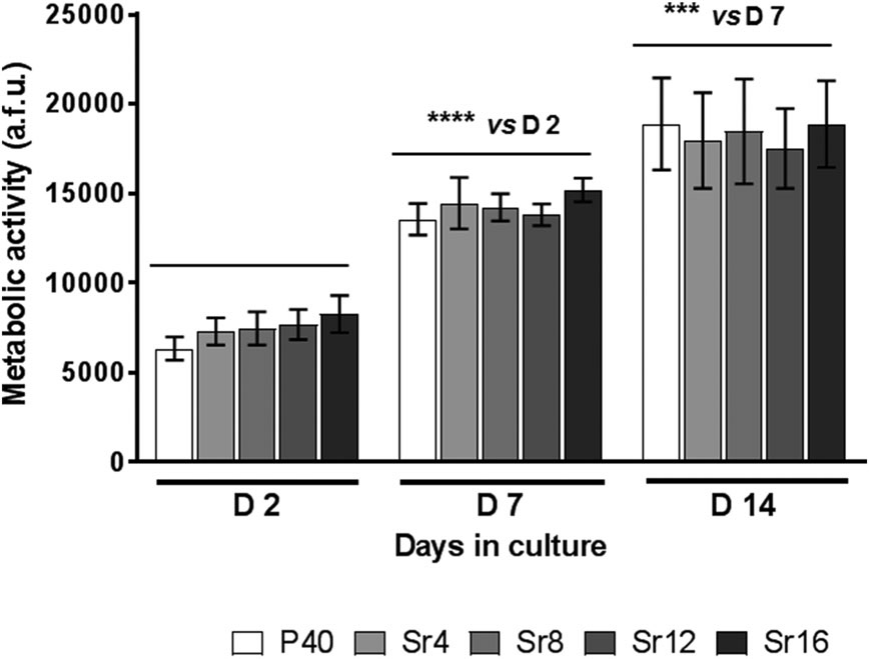
Quantification of metabolic activity in human mesenchymal stem cells at Days 2, 7, and 14 of culture onto phosphate‐based glass microspheres containing varying Sr mol%. a.f.u.: arbitrary fluorescence units; TCP: tissue culture plate. Error bars represent standard error of mean, *n* = 12. ^***^ *p* < 0.001, ^****^ *p* < 0.0001

The metabolic activity of hMSCs cultured on microspheres manufactured from each PBG composition was assayed at Days 2, 7, and 14 after seeding. The results showed a significant increase in cell response over time (*p* < 0.0001), whereas no differences between cell cultures on the different PBG compositions were detected at each time point (*p* > 0.90; see Figure 2).

### 3.3 ALP activity

ALP activity was measured as an early marker of osteogenic differentiation in MG63 and hMSCs after 14 days of culture on both PBG discs and microspheres, respectively.

**Figure 3:**
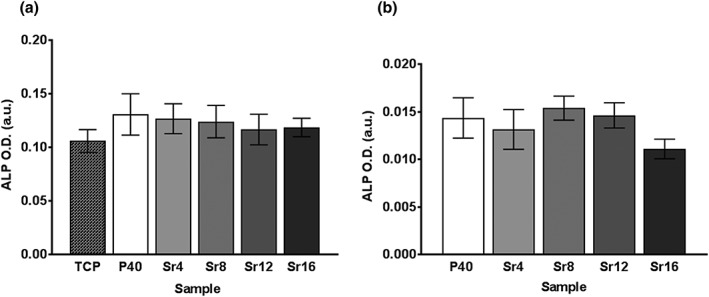
Alkaline phosphatase (ALP) activity measured at Day 14 in (a) MG63 cells cultured on phosphate‐based glass discs and tissue culture plastic (TCP) and (b) human mesenchymal stem cells cultured on phosphate‐based glass microspheres. Graphs show arbitrary units (a.u.) of optical density (O.D.). Error bars represent standard error of mean; *n* = 12

MG63 cells cultured on discs showed no significant difference in ALP activity across all the formulations tested in comparison with the TCP control (*p* > 0.8; see Figure 3a).

ALP activity in hMSCs cultured on PBG microspheres was evaluated at Day 14, and this analysis also showed no significant difference between all compositions (*p* > 0.2; see Figure 3b), in line with the results obtained for MG63 cells.

### 3.4 MG63 cell imaging on discs

**Figure 4:**
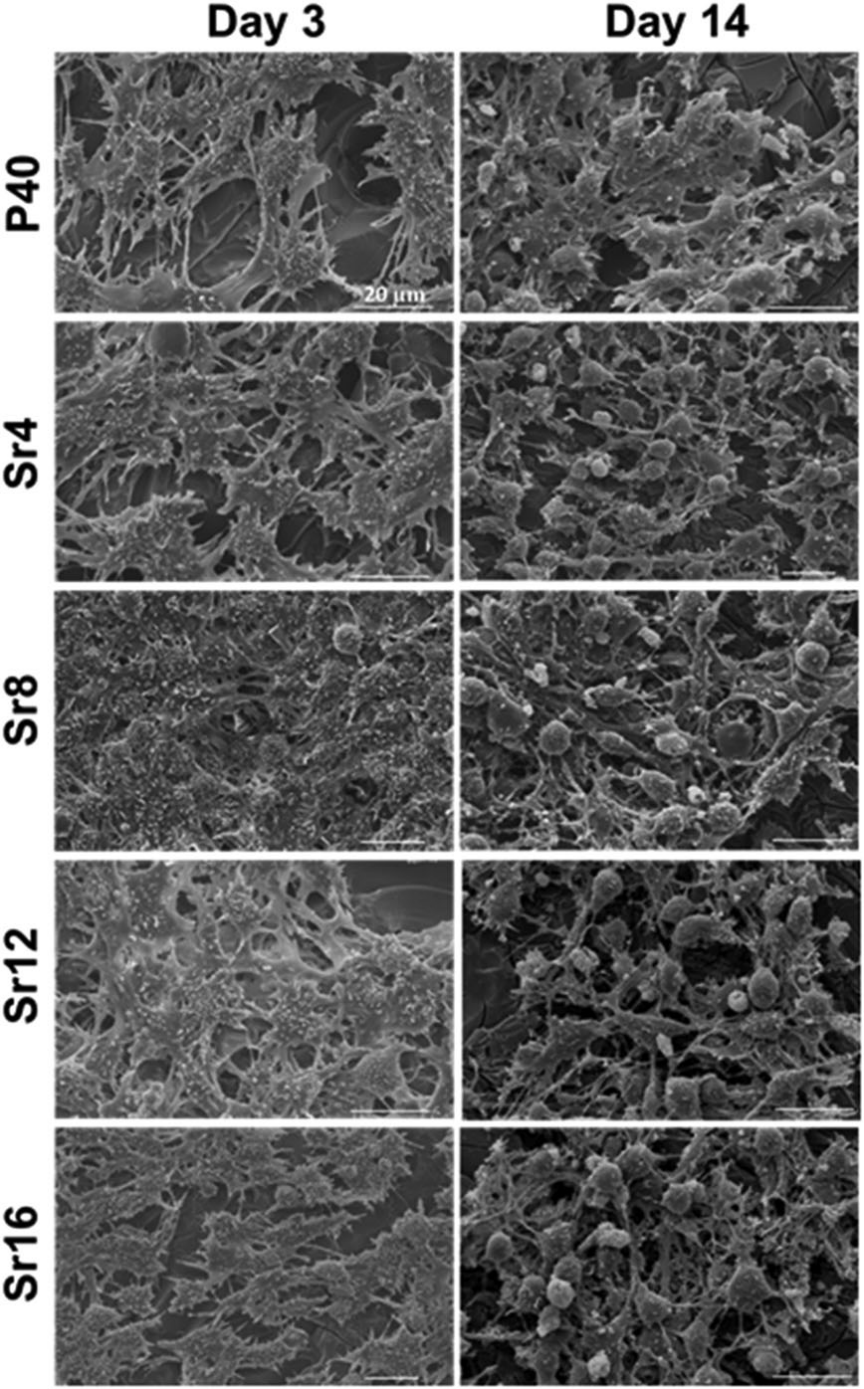
Representative scanning electron microscopy images of MG63 cell cultures on all compositions investigated of phosphate‐based glass discs at 3 and 14 days. Scale bar = 20 μm

MG63 cells cultured on PBG discs were visualised via SEM at Days 3 and 14 (Figure 4). Cells were seen to have attached and spread by Day 3, and a confluent layer across all sample compositions was observed at Day 14. Cells appeared to be spindle‐like with lamellipodia and filopodia extending to neighbouring cells in all glass formulations studied.

### 3.5 hMSC cell imaging on microspheres

**Figure 5:**
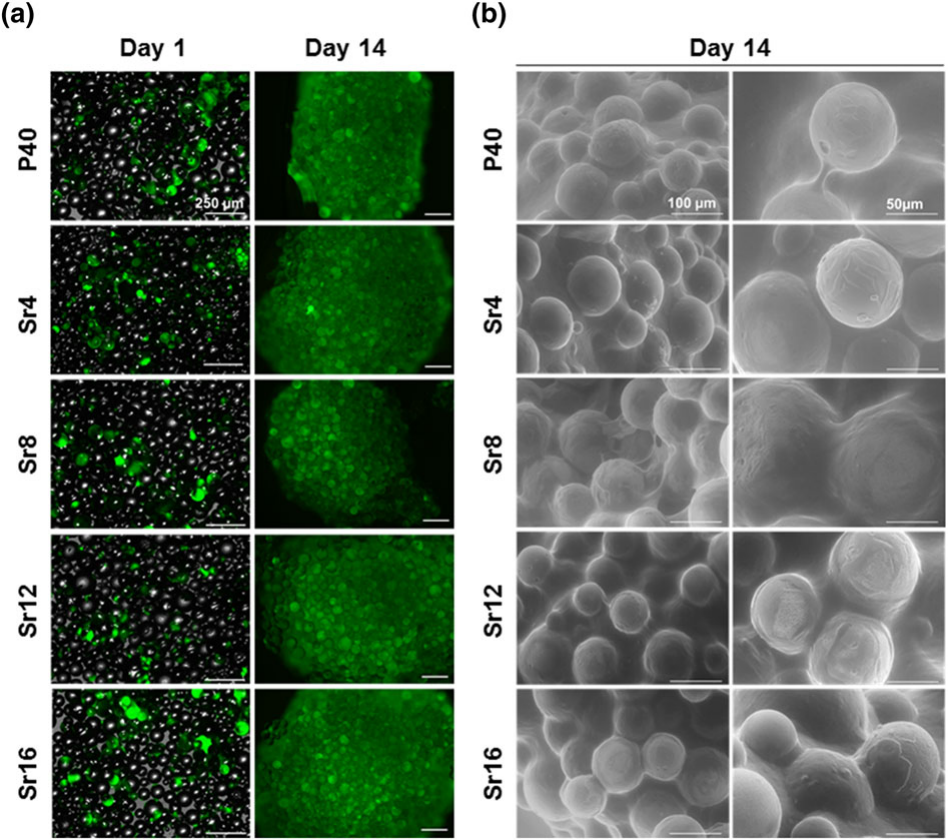
Representative images of human mesenchymal stem cells seeded on phosphate‐based glass microspheres through (a) live cell fluorescence imaging and (b) environmental scanning electron microscopy. Scale bar: a = 250 μm; b = 100 and 50 μm [Colour figure can be viewed at wileyonlinelibrary.com]

Live fluorescence images of hMSCs showed a similar level of cell adhesion to PBG microspheres across all formulations at Day 1, whereas the presence of cell–microsphere aggregates was observed at Day 14 (see Figure 5a). These aggregates were further analysed via ESEM imaging, which showed the attachment and spreading of cells on the microsphere surface, as well as the presence of a dense cell‐derived matrix surrounding the microspheres (see Figure 5b).

## 4 DISCUSSION

This study aimed to assess the biological properties of a series of near invert PBGs where CaO content was gradually substituted for SrO by 0% to 100% (Patel et al., 2017) and manufactured in disc and microsphere forms. Microspheres in particular provide the advantage of uniformity in size and shape, making delivery to the target tissue possible through simple injection procedures (Kim & Pack, 2006). Spherical material morphologies also enable enhanced versatility for filling even complex defect shapes compared with bulk scaffolds, which tend to have predetermined shapes (Choi, Zhang, Yeh, Wooten, & Xia, 2012). The increased surface area also allows for efficient cell attachment and spreading, whereas the microsphere interparticulate gaps would be beneficial for oxygen and nutrient flow (Cai, Chen, Hong, Liu, & Yuan, 2013). MG63 cells have been widely used as a versatile tool for the evaluation of the osteogenic potential of new materials and compounds (Rodriguez, Saxena, Hixon, Sell, & Bowlin, 2016), whereas hMSCs cells have been included in this study as a clinically relevant cell model, largely used in in vitro and in vivo studies of bone regeneration (Knight & Hankenson, 2013).

As previously demonstrated, the gradual replacement of CaO content for SrO by 0% to 100% in the glass system used in the this study (40P_2_O_5_·(16‐x)CaO·20Na_2_O·24MgO·xSrO, where x was 0, 4, 8, 12, and 16 mol%) did not significantly alter the PBG dissolution rate, providing a means of controlled release of therapeutic ions such as strontium, magnesium, phosphate, and calcium (Patel et al., 2017). The evaluation of cytocompatibility performed in this study showed that all PBG formulations, manufactured as discs or microspheres, supported cell adhesion and growth up to 14 days with no major differences observed between the formulations investigated. Also, in the case of MG63 cells, the cell response was lower than the TCP control, whereas these differences were not observed from the DNA quantification analyses. However, in the literature, contrasting data are available. The incorporation of 5 and 10 at% of SrO into hydroxyapatite‐induced similar levels of proliferation of MG63 cells than the TCP at Days 14 and 21 (Boanini et al., 2014). On the contrary, all formulations of an invert glass system with 0 to 100 mol% of SrO substitution for CaO (30P_2_O_5_·(60‐x)CaO·7MgO·3TiO_2_·xSrO (where x = 0, 17, 33, 50, 67, 83 and 100 mol%) induced a significant increase in cell number of MC3T3‐E1 cells in comparison with TCP control at Days 3 and 5 (Lee et al., 2015). Interestingly, the Bioglass series 46.46SiO_2_·1.07P_2_O_5_·26.38Na_2_O·23.08CaO with 0, 10, 50, and 100 mol% of CaO substitution for SrO significantly induced the metabolic activity of Saos‐2 cells in comparison with the SrO‐free formulation (Gentleman et al., 2010). A dose‐specific induction of proliferation was reported for MG63 cells grown on PBG containing 1 mol% SrO in comparison with 3 and 5 mol% and the SrO‐free samples (Abou Neel et al., 2009; N. Lakhkar, Abou Neel, Salih, & Knowles, 2011). All these data suggest that the substitution of CaO for SrO is cytocompatible in a wide range of glass formulations including those selected in this study.

The SEM and fluorescence image micrographs obtained in this study complemented the cell metabolic activity showing cell adhesion to the glass surface and spreading from early time points for both cell types, with no obvious difference in cell morphology with varying concentrations of SrO in the PBG formulations investigated. The influence of glass composition and degradation rate on cell behaviour was discussed by Skelton et al. (2007) who demonstrated the lack of attachment, proliferation, and increased cell death for human bone marrow stromal cells and human fetal osteoblasts cultured on ternary PBG systems. Such detrimental effects on cell behaviour were attributed to an unstable PBG surface in contact to cell culture medium, which led to a rapid dissolution of these fast degrading ternary formulations preventing cells from adhering to the surface. In vivo too, the degradation rate of a material can significantly affect the outcome of the therapeutic strategy by either altering the structural support provided to the endogenous tissue and/or stimulating the inflammatory response (Vishwakarma et al., 2016; Yu, Tang, Gohil, & Laurencin, 2015).

The dissolution behaviour of the PBG formulations studied here has been previously reported, showing a more stable PBG surface with the addition of SrO, with a weight loss ranging between 0.2% and 0.13% in 30 days for the discs (Patel et al., 2017). Evidence also suggests that the degradation rate of the glass increases with spherical geometries (Hossain et al., 2018; Islam, Hossain, Sharmin, Parsons, & Ahmed, 2017); however, in vivo studies would need to be conducted to establish how this could affect the regenerative potential of the material.

The ability of a biomaterial to induce osteogenic differentiation is a desirable property in view of the application for bone repair. In this study, the assay of ALP activity was performed as an early marker of osteogenic commitment. This enzyme is constitutively active at low levels in all the cells, but its activity significantly increases during the early stages of osteogenic differentiation; therefore, it is commonly considered a good marker for the detection of early osteogenic differentiation (Golub & Boesze‐Battaglia, 2007; Prins et al., 2014; Stein, Lian, & Owen, 1990). Strontium ranelate, an Sr‐based drug previously administrated to osteoporotic patients, has been reported to have a dual role by enhancing differentiation of osteogenic progenitor cells as well as by inhibiting osteoclastogenesis (Brennan et al., 2009; Fonseca & Brandi, 2010). In this study, no significant difference in ALP activity was observed between all the formulations including the TCP control with either cell types. In the literature, different PBG systems containing a wide range of SrO mol% have been reported to promote ALP activity. For instance, Lee et al. (2015) found a dose‐dependent response in the induction of ALP at Day 10 using mouse MC3T3 cells cultured on the invert glass system 30P_2_O_5_·(60‐x)CaO·7MgO·3TiO_2_·xSrO (where x = 0, 17, 33, 50, 67, 83, and 100 mol%). Also, higher ALP activity of MG63 cells was observed at Day 14 of culture on PBG glass discs with 17.5 mol% of SrO (Al Qaysi et al., 2015). Incorporation of 5 and 10 at% of SrO into other biomaterials such as hydroxyapatite resulted in significant increase of ALP activity compared with the TCP control in a dose‐dependent manner (Boanini et al., 2014). It seems that such osteogenic effect exerted by strontium may be mediated by the activation of the Wnt/β‐catenin pathway both in vitro and in vivo (Yang et al., 2011), by stimulating the activity of the ALP enzyme as well as the production of ECM components including collagen type‐I and osteopontin. It is conceivable that the observed response may be the result of a combination of factors rather than the sole exposure to strontium including the combination and rate of ions simultaneously released during glass degradation, which have all been described to influence cell behaviour (Maeno et al., 2005).

Overall, the findings of this study complemented previous works, which aimed to characterise the effect of gradually substituting CaO for SrO in a PBG system. More interestingly, our group has recently reported the manufacturing of PBG microspheres in highly porous conformation with larger surface area and increased degradation rate (Hossain et al., 2018). These microspheres have also shown the ability to encapsulate stem cells and will be further investigated for promoting cell growth and pore colonisation, to deliver enhanced advantages for bone tissue growth via a biomimetic environment. Future work will also be aiming to manufacture novel formulation of porous PBG including therapeutic ions such as strontium with the aim to provide new insights for the design of novel materials for minimally invasive orthopaedic repair and regeneration applications. A comprehensive evaluation of the biological potential of these formulations in vitro would provide important additional information for the further progression toward in vivo studies. In particular, in view of the anti‐resorptive activity attributed to strontium as well as the importance of appropriate vascularisation to support the new tissue, further studies could evaluate how these glass formulations can affect the activity of osteoclast cells and the formation of vessel‐like structures by endothelial cells.

## 5 CONCLUSIONS

This study showed that discs and microspheres manufactured from the near invert glass system 40P_2_O_5_·(16‐x)CaO·20Na_2_O·24MgO·xSrO (x = 0, 4, 8, 12, and 16 mol%) are cytocompatible and promoted adhesion and growth of human MG63 and hMSCs, respectively, up to 14 days. The observations made in this study suggest that these materials are appropriate for the culture, expansion, and cellular activity of human cells constituting as excellent candidates for bone regeneration.

## CONFLICT OF INTEREST

The authors declare that they have no conflict of interests.

## AUTHOR CONTRIBUTIONS

1. L. Macri‐Pellizzeri: Study design; acquisition, analysis and interpretation of cell culture data; conception and drafting the manuscript
2. U. Patel: Study design; acquisition, analysis and interpretation of cell culture data; conception and drafting the manuscript
3. K. M. Z. Hossain: Study design; materials manufacture and characterisation assistance
4. B. E. Scammell: Critical discussion of the acquired data for clinical applications
5. D. M. Grant: Critical discussion of the materials characterisation data
6. C. A. Scotchford: Revision and discussion of the acquired cell culture data
7. A. C. Hannon: Materials structural characterisation assistance
8. A. R. Kennedy: Materials characterisation assistance
9. E. R. Barney: Materials structural characterisation assistance
10. I. Ahmed: Study conception; critical revision and discussion of the drafted manuscript; approval for submission
11. V. Sottile: Study design; revision and discussion of the drafted manuscript; approval for submission
